# Single Cell Analysis of Drug Distribution by Intravital Imaging

**DOI:** 10.1371/journal.pone.0060988

**Published:** 2013-04-10

**Authors:** Randy J. Giedt, Peter D. Koch, Ralph Weissleder

**Affiliations:** 1 Center for Systems Biology, Massachusetts General Hospital, Boston, Massachusetts, United States of America; 2 Department of Systems Biology, Harvard Medical School, Boston, Massachusetts, United States of America; University of Navarra, Spain

## Abstract

Recent advances in the field of intravital imaging have for the first time allowed us to conduct pharmacokinetic and pharmacodynamic studies at the single cell level in live animal models. Due to these advances, there is now a critical need for automated analysis of pharmacokinetic data. To address this, we began by surveying common thresholding methods to determine which would be most appropriate for identifying fluorescently labeled drugs in intravital imaging. We then developed a segmentation algorithm that allows semi-automated analysis of pharmacokinetic data at the single cell level. Ultimately, we were able to show that drug concentrations can indeed be extracted from serial intravital imaging in an automated fashion. We believe that the application of this algorithm will be of value to the analysis of intravital microscopy imaging particularly when imaging drug action at the single cell level.

## Introduction

Since the advent of intravital imaging, it has been possible to perform single cell and population analysis of tumor biology *in vivo*. In recent years, these methods have also been adapted to the study of drug pharmacology [1], [2], which has been in part enabled by commercially available compounds as well as a growing number of fluorescently labeled therapeutic companion drugs [3]–[6]. To fully realize the potential of this imaging approach, however, and to maximize the data that can be mined in a reasonable time frame, it will be necessary to overcome several challenges. Specifically, intravital imaging videos typically display (i) dense cell fields made up of multiple layers of cells; (ii) cells presenting heterogeneous fluorescent intensity due to differences in the Z location of the cells and to stochastic biological processes; and (iii) movement artifacts due to cellular movement in three dimensions (3-D) as well as to displacement of the anesthetized animal being imaged [7], [8]. Due to these unique features, segmentation of intravital imaging data is challenging, requiring advanced image processing techniques.

A number of different image segmentation methods have been reported. Examples commonly used in cell-based applications include edge detection, watershed-based methods, as well as others. Perhaps the most widely used method for cell segmentation is the relatively simple process of thresholding, where an algorithm is used to separate foreground and background pixels based on the differences between the two classes. This paper therefore focuses on thresholding methods due to their simplicity, their implementation, and their already widespread use amongst biologists.

Within thresholding methods, a number of approaches have already been described [9], including: (i) histogram shape-based methods which function by analyzing histogram peaks and curves; (ii) clustering based methods that divide pixels into two groups, foreground and background, via analysis of global intensity values, to create a segmented image (iii) entropy based methods which utilize the entropic properties of the image foreground and background to segment the image, (iv) object attribute methods that look for commonalities among certain object features for image segmentation; (v) spatial methods that binarize images based on advanced correlations/statistical methods focusing on properties of pixels; and (vi) locally adaptive methods which utilize local information to threshold images in subgroups of local neighborhoods. For segmenting cells and thresholding microscopic images, each of these strategies have advantages depending on the data quality and data type required [7]. Otsu's method [10] is perhaps one of the most common thresholding techniques, and represents an example of a clustering method that functions by thresholding the gray levels of an image into two distinct segments via minimization of variance in each respective group. This technique works most effectively on images where the fluorescent target of interest is relatively uniform in brightness and where the background is similar across the whole of the image; unfortunately, this is not always the case during time lapse imaging of intravenously administered fluorescent drugs. Huang's method [11] is an example of an object attribute method, where in this case, the attribute of interest is the object “fuzziness” measure. Ray's method [12] is an example of an iterative, locally adaptive thresholding method with only three inputs: the number of iterations for determining the threshold, the “power” (a single adjustment that determines the selectivity of the thresholding sequence), and a termination condition setting denoted as “epsilon”. The primary drawbacks of this method are (i) due to its iterative nature, it is computationally intensive; and (ii) unlike other completely automated alternatives, it still requires a certain degree of user input.

The overall goal of the present study was thus to (i) determine a suitable thresholding method for broad use in segmentation algorithms used for intravital microscopy; (ii) utilize a standard thresholding method to create a robust, semi-automated segmentation and data mining algorithm for analyzing pharmacokinetic data at the single cell level in *in vivo* images; and (iii) verify the function of this algorithm. To validate the method, we chose to use videos of a fluorescently labeled poly ADP ribose polymerase (PARP) inhibitor. PARP inhibitors are currently under investigation for use on tumors with BRCA deficiencies, where pharmacokinetics is a key issue [13]–[15]. Ultimately, the ability to collect semi-automated data from *in vivo* images would be of considerable benefit to the pharmacokinetic analysis of fluorescently labeled drugs. Semi-automatic data acquisition would not only provide a non-biased means of assessing drug distribution in cells but would permit the acquisition of larger data sets than otherwise possible through manual delineation of cell borders.

## Materials and Methods

### Tumor model

The human fibrosarcoma cell line HT-1080 was purchased from ATCC (Manassas, VA). Cells were grown in Dulbecco's Modified Eagle Medium (DMEM) supplemented with 10% fetal bovine serum (FBS) and 1% penicillin-streptomycin.

### Reporter cell lines

Histone 2B-red fluorescent protein (H2B-apple) was used to identify the nuclei of individual cancer cells since this model has previously been shown to be robust *in vivo* [2]. pmApple-N1 (Myo1E-pmApple-C1, Addgene, Prof. Christien Merrifield [16]) was cloned by ligating Apple into pmCherry-N1 (Clontech) using AfeI and BsrG1 restriction enzymes. The pTag-H2B-Apple construct was generated by subcloning mApple from pmApple-N1 into pTag-H2B-BFP (Evrogen) using AgeI and NotI. Correct insertion of Apple was confirmed by sequencing the insert in its entirety.

pTag-H2B-Apple was transfected into HT1080 cells using the X-tremeGENE HP transfection reagent (Roche), and followed by selection in 500 µg/ml G418. Single clones were screened for H2B-Apple expression by fluorescence microscopy. Multiple clones showing high fluorescence were selected by fluorescence activated cell sorting, and the brightest 5% of cells were then isolated for expansion. Cells were maintained in Minimum Essential Medium (MEM) supplemented with 10% FBS, 100 I.U. penicillin, 100 µg/ml streptomycin, 2 mM L-glutamine, non-essential amino acids and 100 µg/ml G418.

### Intravital microscopic imaging

All animal experiments were carried out in accordance with guidelines from the Institutional Subcommittee on Research Animal Care. Nude mice (Cox7, Massachusetts General Hospital) were surgically implanted with a dorsal skin window chamber. Approximately 3–4×10^6^ cells, suspended in 1∶1 phosphate buffered saline (PBS) and Matrigel (BD Biosciences, Franklin Lakes, NJ), were implanted under the fascia and allowed to grow for ∼2 weeks. As soon as the tumors became vascularized and had reached 1–2 mm in size, mice were anesthetized with 2% isoflurane in 2 L/minute oxygen on a heated microscope stage. They were then injected via tail vein catheter with either Angiosense-680 (Perkin Elmer, Waltham, MA) or 250 µg of a 500 kDa amino-dextran labeled with Pacific Blue N-Hydroxysuccinimide (NHS) ester (Invitrogen, Grand Island, NY), according to the manufacturer's instructions. Vascularized regions of interest in the tumor were identified by the vessel probe and by the H2B-Apple tumor signal; regions with minimal out-of-plane vessels were chosen for imaging. Time-lapse imaging was initiated prior to injection of the drug. The drug was formulated by dissolving 7.5 µL of a 1 mM solution in dimethyl sulfoxide (DMSO) together with 30 µL of a 1∶1 dimethylacetamide (DMAc)∶solutol solution. 112.5 µl PBS was then slowly added with sonication to obtain a final injection volume of 150 µL.

Static and time series images were collected using a customized Olympus FV1000 based on a BX61-WI confocal microscope (Olympus America). A XLUMPLFLN 20× water immersion objective (NA 1.0, Olympus America) was used for data collection. BODIPY-FL, H2B-Apple, and vascular probes were scanned and excited sequentially using a 405 nm, a 473-nm, a 559-nm and/or a 633 nm diode laser, respectively, in combination with a DM405 488/559/635-nm dichroic beam splitter. Emitted light was then separated and collected using appropriate combinations of beam splitters (SDM473, SDM560, and/or SDM 640) and emission filters BA430–455, BA490–540, BA575–620, BA575–675, and/or BA655–755 (all Olympus America). Control tumors were used to determine good settings for voltage and laser power, and to optimize imaging conditions by ensuring that no photobleaching or phototoxicity occurred with the imaging settings used. Time-lapse movies were corrected for small shifts using the StackReg plugin in ImageJ (vers. 1.47a). Typically, images were acquired every 75 seconds for the duration of experiments.

### Thresholding Survey and Algorithm Implementation

A survey of thresholding images was initially conducted either using the freely available image processing software ImageJ (National Institutes of Health, Bethesda, MA) or by implementation into Matlab (Mathworks, Natick, MA). Promising methods were then implemented into an image-processing algorithm in Matlab. Quantitative comparisons were created manually using the freeware GNU Image Manipulation Program (GIMP, Groton, MA) by two independent reviewers (Supplemental Fig. 1). Comparisons were applied to both manual images and the results are presented as averages.

### Fluorescent Drug

A solution of boron-dipyrromethene-fluorine (BODIPY-FL) succinimidyl ester (5.0 mg, 12.8 µmol; Invitrogen, Carlsbad, CA) in acetonitrile (250 µL) was reacted with a solution of 4.7 mg (12.8 µmol, dissolved in 250 µL acetonitrile) of 4-[[4-fluoro-3-(piperazine-1-carbonyl)phenyl]methyl]-2*H*-phthalazin-1-one [17] in the presence of triethylamine (4.6 µL, 64.2 µmol). The two components reacted together cleanly within 4 hours at room temperature. The product AZD2281-BODIPY FL was then isolated using standard high-performance liquid chromatography (HPLC) techniques performed on a Waters (Milford, MA) liquid chromatography–mass spectrometry (LC-MS) system and using a Waters XTerra® C18 5 µm column. The title compound was isolated in 72% yield and its identity confirmed using LC/MS, HPLC, nuclear magnetic resonance (NMR) and high resolution mass spectrometry (HRMS) techniques. The compound had a maximum absorbance at 507 nm and a maximum emission at 527 nm. This compound has previously been shown to retain similar bioactivity to the unmodified drug [18].

### Statistical Analysis

For data analysis, values for each of three individual measurement methods were compiled and ranked following evaluation of six individual images (for additional images used in the statistical analysis see Supplemental Fig. 2). After ranking, an analysis using Friedman's rank sums test was used to evaluate whether any statistical differences were present among the distributions. For sets where a difference was detected, the Wilcoxon rank sum test was used as a pairwise test to determine group subset differences. The *p* values<0.05 were considered significant.

## Results

### Thresholding Method Comparison and Selection

**Figure 1:**
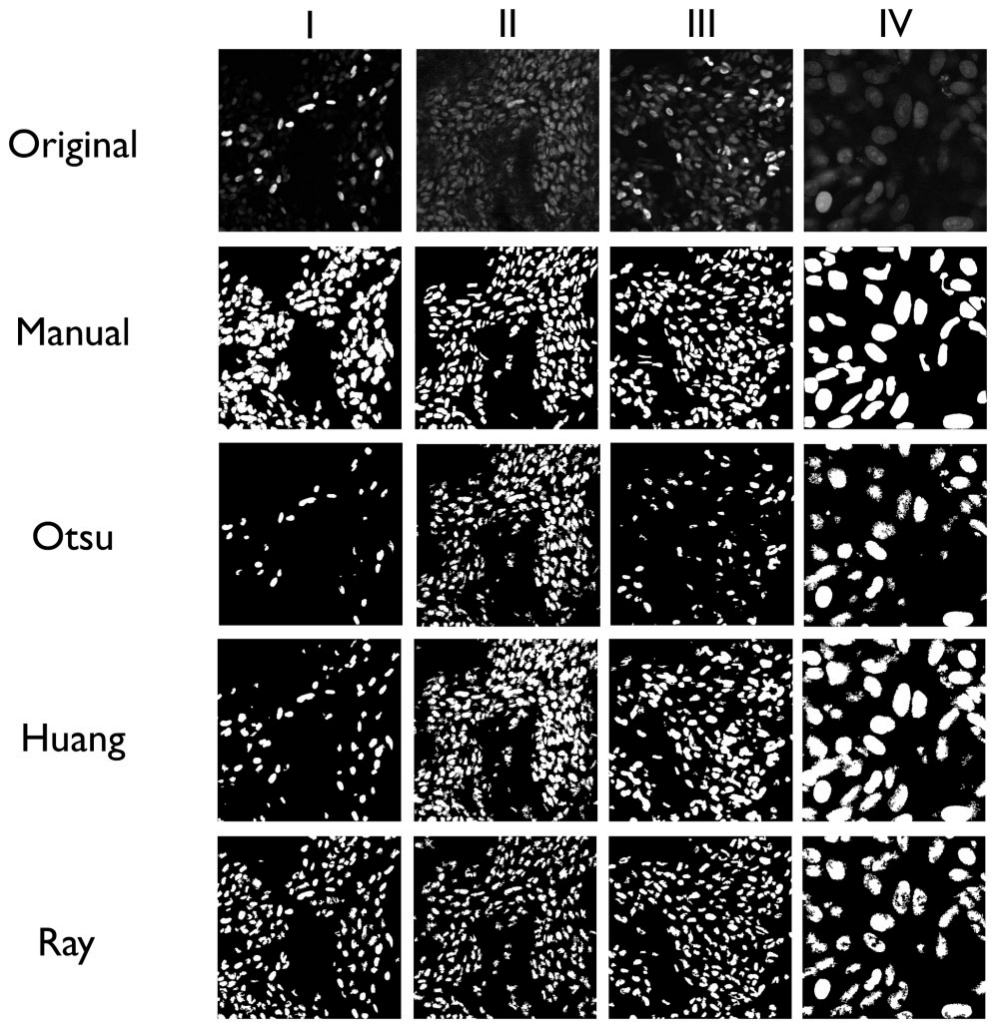
A diverse set of typical intravital images was analyzed using different thresholding methods to determine their suitability for cell segmentation across a variety of conditions. Cell nuclei are shown at various magnifications labeled with H2B-Apple. I. An image displaying multiple fluorescent brightness levels. II. A dense cell field image. III. A dense cell field with multiple fluorescent brightness levels. IV. A high magnification image with intracellular details. The techniques analyzed were: manual thresholding, Otsu's method, Huang's method, and Ray's method.

We initially surveyed a variety of thresholding algorithms using sample pharmacokinetic data (Table 1). Promising methods, including a clustering based method (Otsu), an object attribute method (Huang), and a locally adaptive method (Ray), were then further analyzed against a range of intravital image data sets. Representative images displaying four different imaging complications (uneven fluorescence expression, a high cell density field, a high cell density field with uneven fluorescence expression, and high magnification images with interior details) were selected. These were subsequently used to quantitatively compare the different thresholding methods. Images obtained using a manual threshold were compared mathematically to individual thresholding methods (see below). Visual comparisons are shown in Figure 1. The overall goal of these quantitative measures was to determine the suitability of each method for pharmacokinetic analysis in intravital imaging. Calculations were thus focused on the amount of correctly classified area as well as on the correctness of the number of regions. These measures seem to be the most appropriate for data acquisition of this type, since they focus on sources of error in intravital pharmacokinetic analysis, such as incorrectly classified background or foreground pixels, the presence of large numbers of artifacts following thresholding, and/or bias against fluorescence intensity heterogeneity.

**Table 1:** Image Thresholding Methods Surveyed.

Method Type	Examples
Histogram	Doyle [31]
	Glasbey [32]
	Tsai [33]
	Zack [34]
Clustering	Kittler [35]
	Otsu [10]*
	Ridler [36]
Entropy	Kapur [37]
	Li [38]
	Shanbhag [39]
	Yen [40]
Object Attribute	Huang [11]*
	Prewitt [41]
Spatial	Beghadi [42]
Locally Adaptive	Ray [12]*

A variety of methods were sampled to identify the most promising techniques for intravital imaging analyses. Those marked with an asterisk (*) were used for further analysis.

Misclassification error (ME) [19] reflects the percentage of background pixels wrongly characterized as foreground, as well as the percentage of foreground pixels wrongly characterized as background. If *B_o_* and *B_T_* are the background pixels of the initial and thresholded images respectively, and if *F_o_* and *F_T_* are the foreground pixels respectively, and if |… | represents the cardinality of the set, ME is described as,
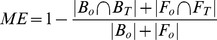



We subsequently calculated a simple measurement of total region number nonuniformity (TRNU) by assuming that *R_o_* is equal to the total number of regions found in the manually thresholded image, and that *R_T_* is the number of regions found in the images following each of the other thresholding methods,
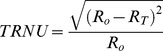



Finally, we were interested in comparing the range of fluorescent intensities in the manually thresholded image to the range of intensities present in each image obtained via the other thresholding methods. To do so, we modified a variance nonuniformity measure (VNU), which typically assumes that well segmented images will have a uniform fluorescent intensity value [20], [21]. This assumption, however, is unlikely to hold true for intravital images containing multiple levels of fluorescent intensity. Our contrast measure was therefore calculated as,
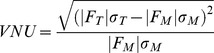
assuming *σ_T_* is the variance of the pixels in the thresholded image, *σ_M_* is the variance of the manually thresholded image and *F_M_* represents the foreground pixels of the manually thresholded image.

**Figure 2:**
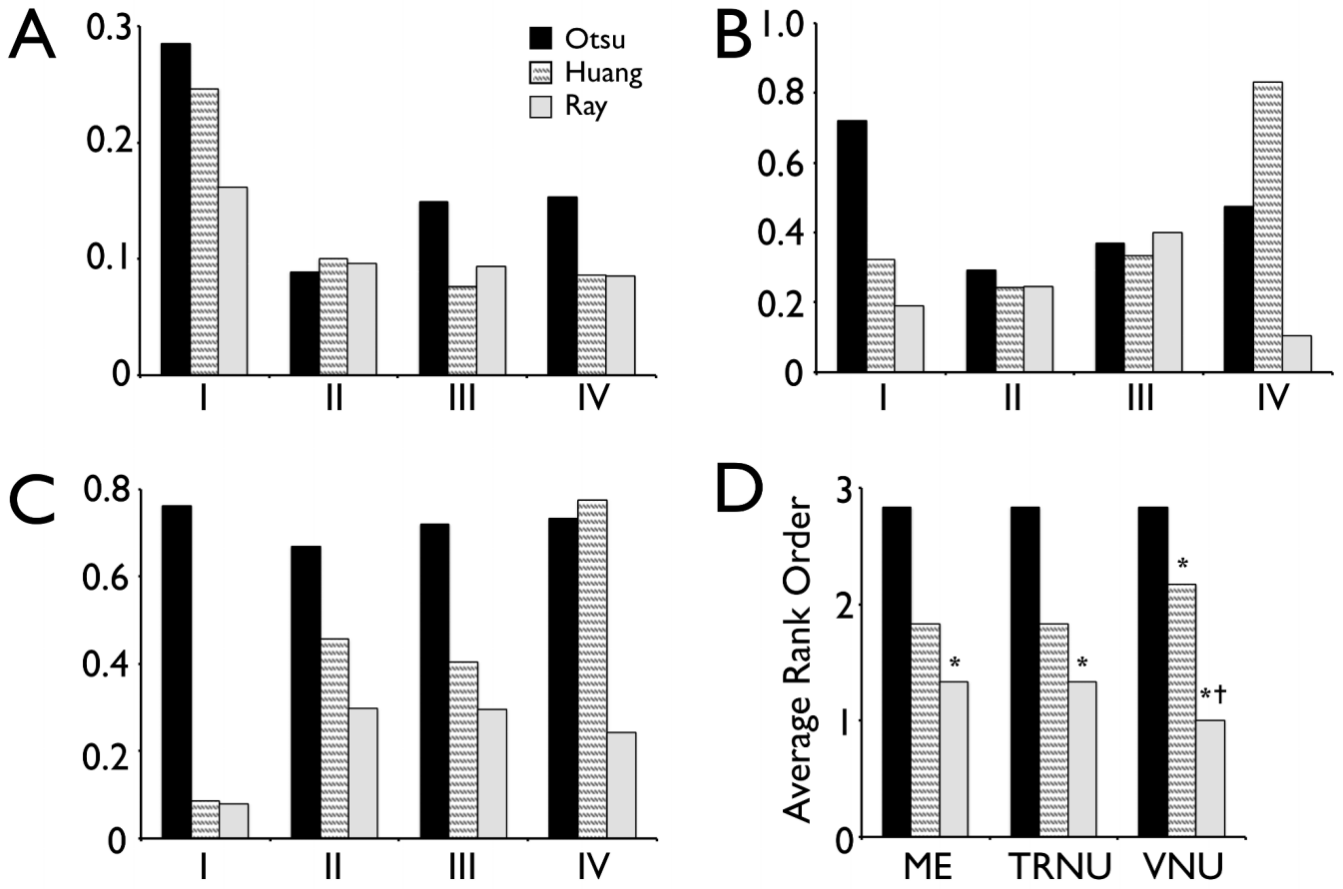
Quantitative comparison of thresholding methods for intravital microscopy. The various thresholding methods described (Otsu, Huang and Ray) were quantitatively compared to determine the best non-biased method(s) for each imaging type. Two independent reviewers created manual images via cell border identification for each image (I–IV in Figure 1). Images obtained with each thresholding method were then compared to the manually thresholded images, and averaged using various measures found in the literature including: A. the misclassification error, which penalizes misclassified foreground and background pixels in each image; B. total region number nonuniformity, which penalizes images based on incorrect numbers of total regions found; C. region variance nonuniformity, which compares the variance of the segmented region fluorescent intensity between manually thresholded images and the images obtained via the other thresholding methods (Otsu, Huang and Ray); D. The average rank order across six typical intravital images (see Supplemental Fig. 2 for additional images) for each measure (ME, misclassification error; TRNU, region number nonuniformity; VNU, region variance nonuniformity). * *p*<0.05 relative to Otsu's method, and † *p*<0.05 relative to Huang's method.

The results of our quantitative comparison between images are presented in Figure 2. Analysis of image I showed that for most measures, Ray's method was superior, or equal, to Huang's method; Otsu's method, however, produced unsatisfactory results for images with multiple levels of brightness. For image II, which consisted of a single level of brightness within a dense cell field, the performance of all thresholding methods was approximately equal. For image III, which consisted of a dense cell field with unequal brightness, the results were similar to image I, whereas the Ray and Huang methods performed at approximately the same level, with Ray's method perhaps performing slightly better. Finally, in image IV, the high magnification image, Ray's method appeared to produce the best results, particularly for identifying outer cellular/nuclear boundaries.

### Algorithm Development

**Figure 3:**
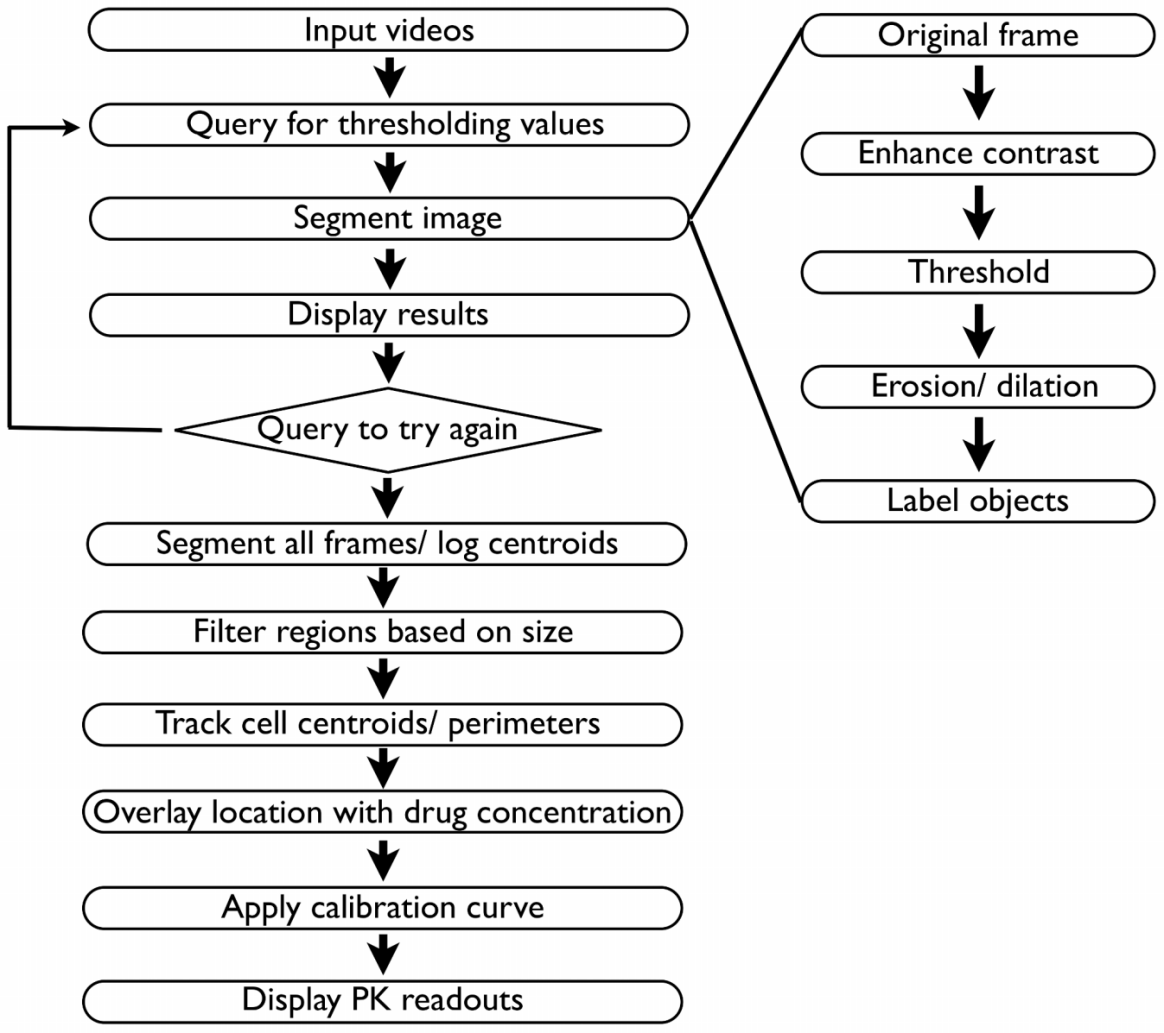
Overview of the image processing method. The left side of the diagram displays the overall proposed algorithm for analyzing intravital images and determining drug concentration. This algorithm is made up of an iterative section that allows the user to generate the best possible segmentation (top) and a processing module that filters through all videos after satisfactory values have been obtained. On the right, the specific segmentation algorithm used in conjunction with the thresholding method is displayed.

To develop a segmentation method (Fig. 3), we began by devising an algorithm that would: (1) create a segmented image with limited user input in an iterative and robust manner; (2) provide secondary filtering of images (including elimination of common defects in thresholded images e.g. image speckling); and (3) segment/label object borders in each image (namely recognize objects, label their borders and log their centroids in preparation for downstream individual object tracking, if desired). By having such features, our goal was to produce an easy-to-use algorithm, capable of analyzing drug concentrations in a relatively heterogeneous range of intravital image types.

The final algorithm is a simple two-step method, which utilizes Ray's method, although other methods such as Otsu or Huang, could easily be incorporated into this structure. To optimize the thresholding and filtering procedure, the user is first asked to define a limited set of variables: frame number to be analyzed, gamma value of the image adjustment, the minimum object size to be considered for analysis, the disk size used for speckling filtering, the number of iterations to run Ray's method, and the power factor to run Ray's method. Once the program applies the desired thresholding function and variables, the results are displayed for subsequent review by the user. The user is then given the option of repeating the analysis or, if the initial results are acceptable, filtering the entire video and obtaining drug properties.

Filtering thresholded images is achieved by using a combination of two filters: a “rough pass” filter (a simple erosion/dilation method that results in the removal of speckling) and a user-defined high-pass filter that eliminates objects too small to be cells. By using two filters, determining the optimal object size to filter in the second portion of the program is thus simplified, since the initial speckling filter eliminates the noisiest elements in the original thresholded image. Image segmentation by first thresholding, and then attenuating the results using a series of simple filters provided excellent program flexibility as well as ease of use.

Overall object segmentation was completed using established image processing functions [22] (see Supplemental Fig. 3 for detailed morphological operations). Briefly, image channels displaying cell locations and drug fluorescence were first separated. In the cell location channel, objects were labeled using standard commands in Matlab, and object centroids/borders were logged for each object in each frame of the video. Drug concentrations were then determined from the drug fluorescence channel by averaging the amount of fluorescence contained within the previously identified object borders.

Fluorescent intensities were converted into drug concentrations via a calibration curve. Specifically, concentrations of the BODIPY-FL labeled drug were diluted in PBS ranging from the nanomolar to micromolar range. Images of the PBS-drug solution were then acquired from each drug concentration utilizing the exact microscope specifications used for animal imaging. In addition, blood vessel concentrations of drug were also correlated with control dilutions created in blood, verifying the calibration. Optimal microscope settings, including laser intensity, were determined via trial experiments in order to minimize possible imaging and signal quantification defects. In general, these settings precluded typical issues such as background saturation of the drug signal or high rates of photobleaching that would significantly alter drug concentration measurements *in vivo*.

### Results of using the Ray Thresholding Method in 3-D Models

**Figure 4:**
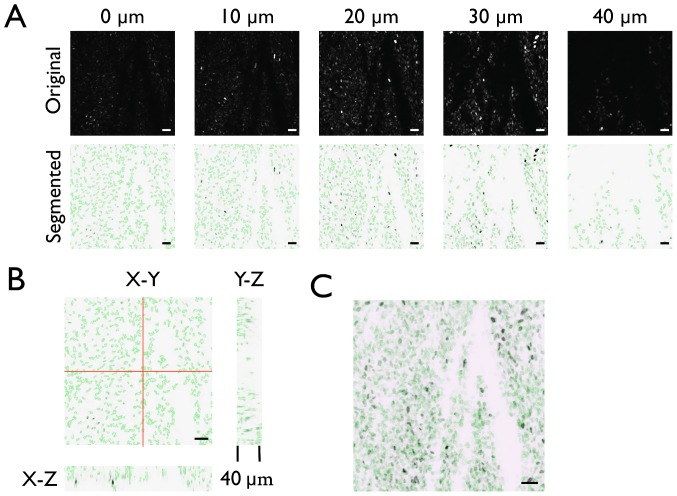
Cell segmentation on a high cell density Z-stack. A. Representative images from a typical intravital imaging Z-stack with H2B-Apple labeled nuclei (top row) and segmented regions identified from a negative of the original image using the described segmentation algorithm (bottom row; green outlines depict the segmented cell regions detected by the algorithm in each Z-slice). All scale bars represent 50 µm. B. Orthogonal views of the 3D Z-stack displaying segmented cell region outlines (green) in each view. C. Summation of the Z-stack containing all combined segmented region outlines (green).

In addition to thresholding individual images, a critical component of tracking objects is to have consistency in results over the course of a data set (i.e., the thresholding method should produce robust results despite various imaging defects occurring throughout a video). Thus, based on its superior performance in our quantitative assessment as well as on its greater flexibility, we chose to analyze results from the previously described algorithm further using 3-D images. By using 3-D images, we were not only able to evaluate the possibility of applying this method to 3-D image analysis but could also determine the consistency of this thresholding method over the course of a time lapse. Figure 4 displays the results of this analysis across a 40 µm intravital imaging Z-stack.

### Drug Concentration and Localization Tracking

**Figure 5:**
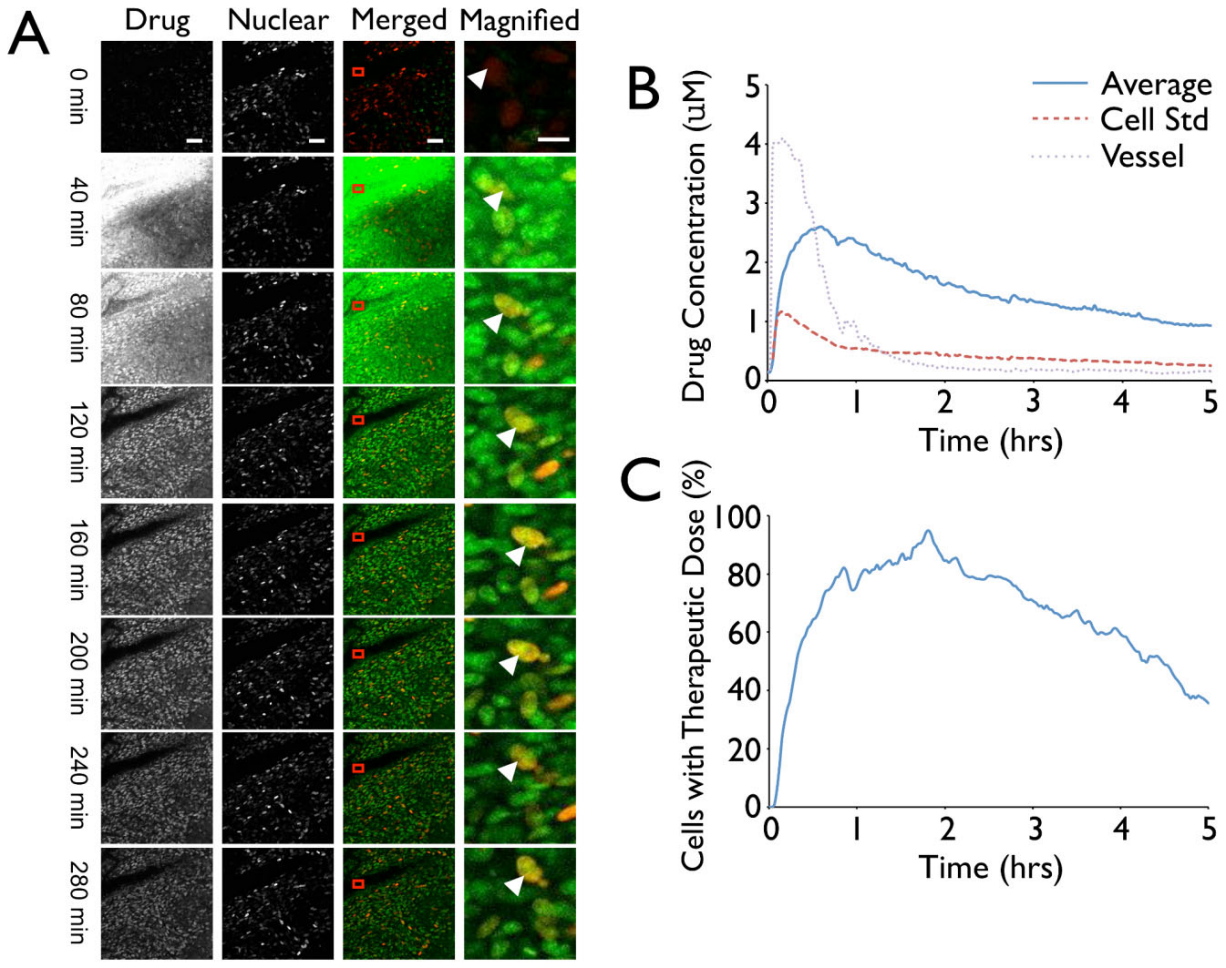
Analysis of average nuclear drug concentrations over time. A. Representative images from a 5 hour PARP inhibitor pharmacokinetics assay. Far-Left Panel: drug distribution. Scale bar represents 50 µm. Middle-Left Panel: H2B Nuclear Marked tumor cells. Scale bar represents 50 µm. Middle-Right Panel: merged images displaying both the drug (green) and tumor cells (red). An area of the closest vessel was also selected to analyze the dynamics of drug distribution through the vasculature (Red box). Scale bar represents 50 µm. Far-Right Panel: Magnified cells from the presented image shown over time. The white arrow indicates a single cell visually tracked throughout the course of the video. Scale bar represents 10 µm. B. The average and standard deviation of nuclear drug concentration in all cells over time was analyzed using the described segmentation algorithm. The vessel concentration dynamics were also analyzed by quantifying drug channel fluorescence within an area of the vessel. C. The number of cells receiving a therapeutic dose of the drug over time.

We next used time-lapse videos of fluorescently labeled PARP inhibitor to automatically extract pharmacokinetic information from intravital images (Fig. 5A). Drug concentrations were logged as the average of each cell's drug concentration, and the standard deviation of the cells; drug concentration in an area of a local vessel is also displayed (Fig. 5B). These analyses also provide rapid answers to broader questions such as what is the fraction of cells with no or subtherapeutic drug concentrations at a given time point, or how much drug is located in the nucleus versus the cytoplasm. Using PARPi as a model, we show that at 2 hours, during the maximum range of drug distribution and intensity, only ∼3% of cells had subtherapeutic levels (defined as a 1.5 µM concentration [23]) (Fig. 5C). In addition, by making an assumption about the approximate size of the cytosol surrounding the nucleus (a simple dilation of the nucleus size) in individual cells and extrapolating this value across all cells, it was estimated that ∼95% of the drug was located in the nuclear compartment at steady state. With the addition of markers of cell membrane and other intracellular organelles, it will be possible to generate more detailed information regarding the behavior of the cytosolic portion of drug distribution over time.

### Single Cell Tracking Over Time

**Figure 6:**
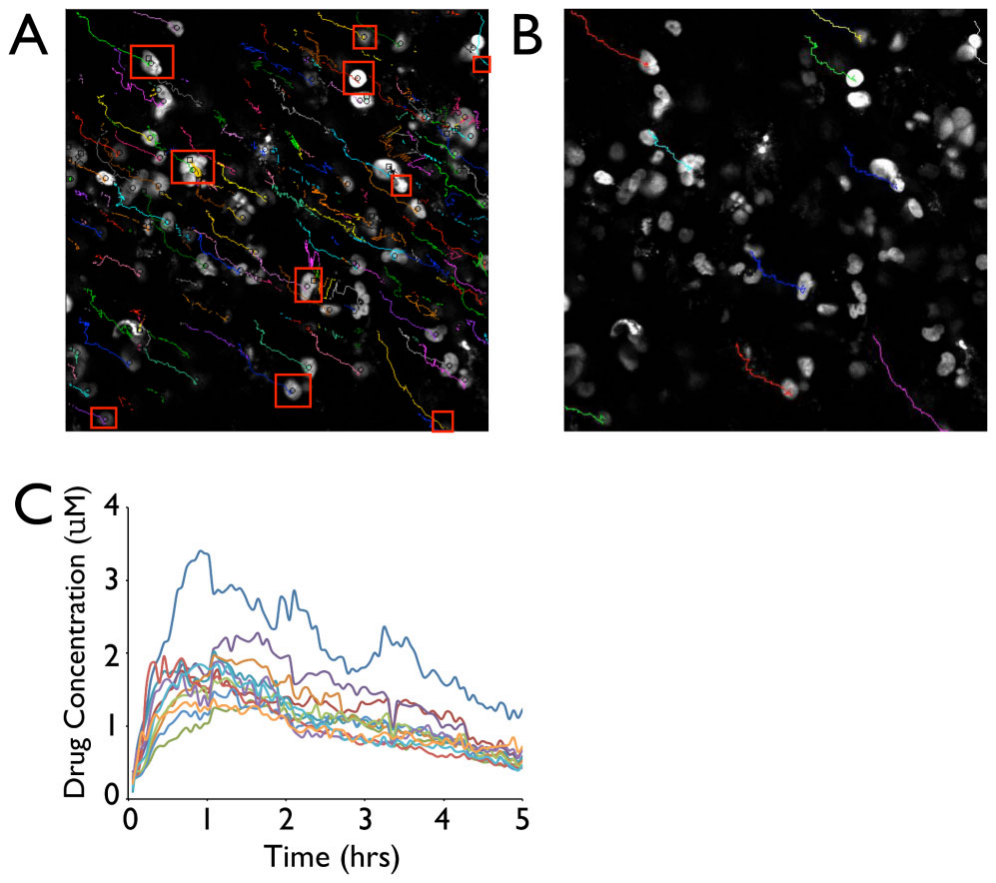
Single Cell Pharmacokinetic Tracking. The segmentation algorithm was combined with a linking program to determine individual cell nuclear drug concentrations. A. The locations of cell nuclei were tracked over 5 hours, using external linking software in a video where both cell movement and image drift were present. Red boxes indicate arbitrarily selected cells used for manual tracking verification of the algorithm. B. Manual tracking of arbitrarily selected cells. C. By combining results using the segmentation algorithm together with the tracking data, drug concentration over time in 10 sample cells could be plotted.

Tumor and host cells are often quite mobile and can move over considerable distances during a several hour imaging session. In addition, despite the best efforts of most current methods of image registration [24], some degree of animal movement may also occur during the course of intravital imaging sessions. This makes the acquisition of consistent data from the same subset of cells challenging. One solution to ensuring the continuity of data is to utilize robust tracking software to log the centroids of cells. The process of tracking cells is normally divided into two steps: (i) Identification of the cell to be tracked in each frame of a video and the logging of their respective centroids (commonly referred to as particle detection) and (ii) Linking these identified cell centroids into coherent cell tracks (commonly referred to as particle linking or tracking). While the detection algorithm presented here can be easily adapted to a variety of freely available linking/tracking algorithms, we formatted our data in this study to be used with the tracking program created by Jaqaman [25]. This program provides several relevant features. First, the software provides a tracking solution for data sets with extremely challenging conditions, such as those with dense cell fields as seen in the images presented in this paper. Second, this program compensates for detection failure, making it an optimal solution for an intravital application where cells may appear or disappear. In addition, although cell division in the time frame of videos presented in this paper are typically not regarded as a problem (due to its relative infrequency) [2], this software allows for merging and splitting events to occur between particles if desired. As described, combining our above-described algorithm with a tracking method has the additional advantage of enabling drug concentration in individual cells to be analyzed over time. This could potentially yield important information regarding chemotherapeutics, where drug resistance in the context of single cells is an area of interest. The results from algorithm cell tracking are presented (Fig. 6A), including 10 manually tracked cell trajectories (Fig. 6B), and 10 sample cells' drug concentrations in a PARP mouse model (Fig. 6C). A search radius maximum of 10 pixels was used for the presented analysis.

## Discussion

Automating microscopic image analysis of time-series (both 2D and 3D) is currently a significant bottleneck in the analysis of *in vivo* drug distribution and function at the population, single cell and intracellular level. In a bid to alleviate this problem, an increasing number of companion imaging drugs are being developed to study how drugs behave and/or fail. By using these drugs in conjunction with orthotopic models [1], [26], advanced motion stabilization techniques [27] and modeling approaches [28], it is anticipated that valuable information regarding the pharmacokinetics and dynamics of drugs will be revealed. Here, we developed and tested an integrated algorithm for automating image analysis with the ultimate output being single cell, intracellular pharmacokinetic data.

To date, a number of thresholding methods have been described. Otsu's method [10] is perhaps one of the most commonly used thresholding methods and is an example of a clustering method that functions by thresholding the gray levels of an image into two distinct segments. By minimizing the weighted sum of the intraclass variances of the foreground and background pixels, an optimal threshold level can be attained. This is because minimizing the intra-class variance is equivalent to maximizing the inter-class variance, which naturally yields the highest contrast between two groups of pixels. In general, Otsu's method works well in situations where images have relatively equal background and foreground pixel numbers i.e., situations where there is a bimodal global distribution of pixel intensities/for bimodal image histograms [29]. Unfortunately, this is not always the case with time-lapse images. Huang's method [11] is an example of an attribute method based on image “fuzziness” levels [30], which are defined as the difference between a gray-scale image and its binary equivalent. This fuzziness measure is used to create a membership function for each pixel in an image. The final threshold of the image is then determined by minimizing the index of fuzziness, as defined by the foreground and background pixel distributions. Object attribute methods generally show improved performance on images where a global threshold proves to be unsatisfactory due to their selection of object features, rather than global intensity levels, in the image. Ray's method [12] is an example of a locally adaptive thresholding method. Locally adaptive methods typically provide superior results to methods proposing global thresholds. In microscopy, locally adaptive methods are ideally suited for use on images with uneven illumination since they depend on local image characteristics rather than on a single global value for determining a threshold. A drawback of these methods, however, is that threshold determination is dependent on a multitude of user inputs (for example, the thresholding window size). This means that the quality of the threshold is dependent upon the results of a trial and error strategy with a wide range of threshold qualities. Consequently, this type of thresholding method can be time-consuming and cumbersome. Ray's method, however, attempts to overcome this problem by proposing a method that iteratively calculates the optimal weighting parameters, which in turn simplifies the thresholding procedure. The main limitation of Ray's method is its current computational expense: the iterative process used to determine the optimal thresholding parameters typically requires approximately 1000 iterations to produce a satisfactory image. As a result, there was a substantial lag time in obtaining thresholded images compared to the other methods assessed in the present study.

In view of its inherent advantages (Fig. 1, 2), we incorporated Ray's thresholding method into our workflow algorithm. To the best of our knowledge, this is the first time that this specific algorithm has been tested and used for cell specific segmentation applications. As demonstrated in this work, the algorithm provides excellent results when dealing with dense cell fields, a scenario where most traditional thresholding methods have the greatest problems. Specifically, the use of a local adaptive method in segmenting cells is helpful in dealing with dense or overlapping cell areas due to its “local” thresholding approach, since cells of interest in such areas typically exhibit multiple levels of fluorescent intensity. In effect, local adaptive methods provide the capability for segmenting multiple levels of cells typical in intravital images, that often appear as a single layer with heterogeneous fluorescence intensity. In these images, overlapping cells will typically be differentiated due to the cells contrast with each other within the small frame of the local thresholded area (Supplemental Fig. 4). Of course, overlapping cells with homogenous fluorescence will require additional processing methods not utilized in this work in order to distinguish individual borders. Overall, this algorithm provides a framework for the analysis of single cell behavior in intravital imaging applications, an emerging area of biological research with currently only very limited methods available. This framework also provides a method of cell segmentation that could be widely adapted to other applications outside of intravital microscopy where data analysis has traditionally proven difficult.

Going forward, the method presented in this report could be expanded in several ways. Firstly, while the segmentation method provided is valid for planar segmentation, accurate 3D fluorescence and drug distribution analysis can only be properly obtained by considering several additional parameters such as high-background components due to tissue scattering, resolving power and confocality of the imaging system, optical aberrations, and detector noise. This is particularly true for imaging modalities such as wide-field and laser scanning microscopy, but is less important for confocal and multiphoton microscopy. Additional work focusing on 3D-assisted segmentation, in combination with image denoising and deconvolution methods, will be the subject of further studies. Secondly, by incorporating information regarding expected cell sizes (or sizes of other parameters representative of intravital images), in the form of filtering mechanisms prior to the thresholding step, the performance of this algorithm could significantly improve. This would also dramatically reduce the number of iterations necessary (using Ray's method) for the production of satisfactory results. Thirdly, adding alternative application routines to this framework (e.g. organelle-specific analysis, assuming an alternative intracellular organelle fluorescence is present) would not only increase the value of this program but would provide additional tools for analysis. Finally, it is likely that by increasing the computational speed of this programming framework, and by incorporating it with on-site microscopy systems, real-time acquisition and analysis of pharmacokinetic (or other application-specific) data could be achieved. Ultimately, this would provide another valuable tool for intravital microscopists.

## Supporting Information

Figure S1 **Comparison of manually thresholded images.** To generate manual thresholding standards for Figure 1, two independent reviewers established manual thresholds by demarcating (to the best of their ability) cell borders in each image. Results from the quantitative assessment of the different thresholding methods described were compared with each of the reviewers' images and the results were averaged.
(TIFF)

Figure S2 **Additional typical intravital images used to perform ranking analysis in** **Figure 2D** **.** Images were analyzed as described for those in Figure 1.
(TIFF)

Figure S3 **Detailed view of overall morphological operations and object labeling on an example image.** This image was thresholded using Ray's method as described, followed by standard morphological operations to remove artifacts produced by the thresholding process. A rainbow color labeled image is presented to show distinct objects recognized by the analysis program.
(TIFF)

Figure S4 **(I–III) Detailed views of cells with heterogeneous fluorescence (indicated by arrows) and segmentation of these areas via the reported algorithm.**
(TIFF)
