# Supplementary figures and images for: Single Cell Analysis of Drug Distribution by Intravital Imaging
Source: PLoS One. 2013 Apr 10;8(4):e60988. doi: 10.1371/journal.pone.0060988 (PMC3622689; doi:10.1371/journal.pone.0060988)

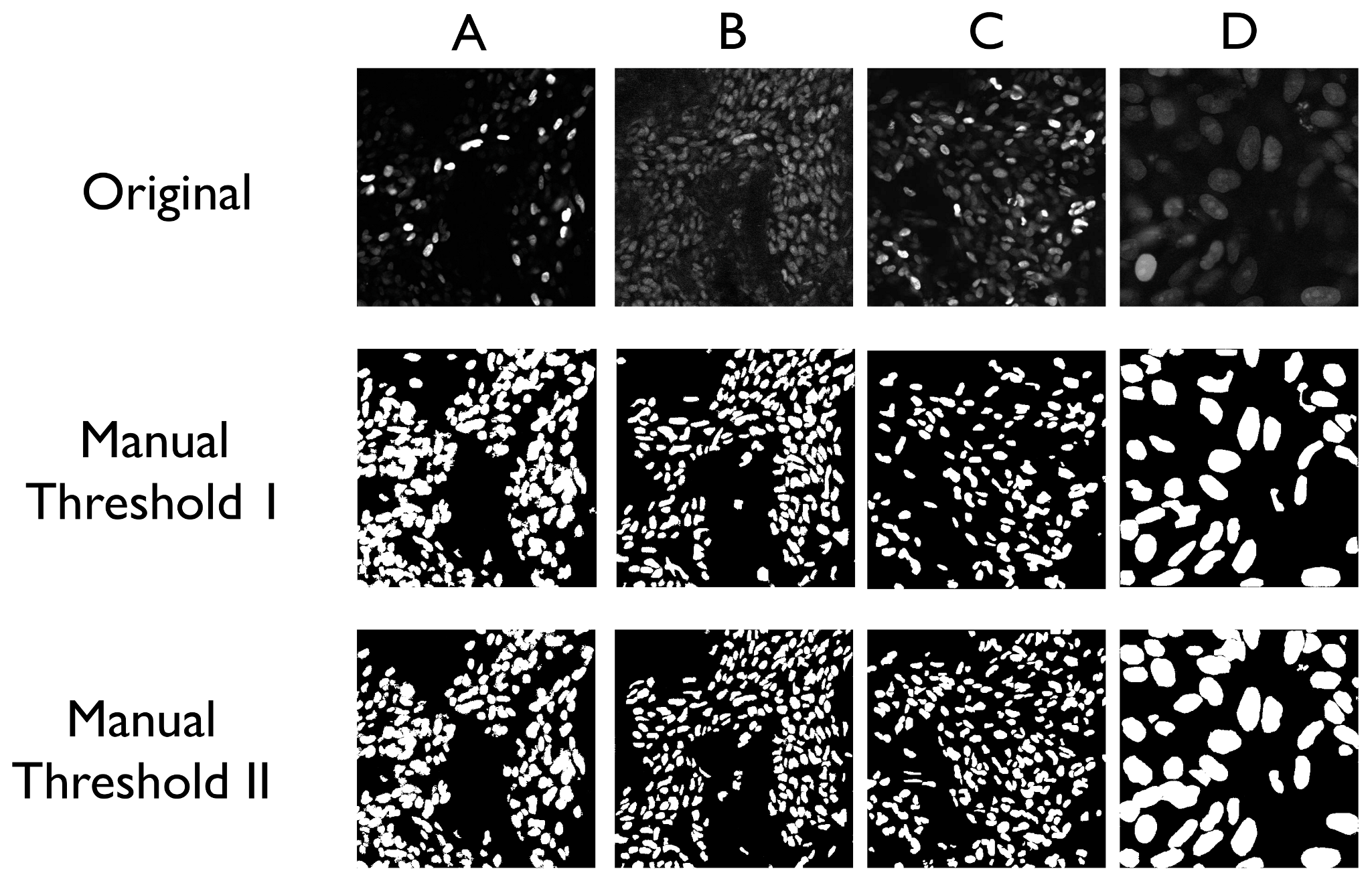

Supplement: Figure S1 — Comparison of manually thresholded images. To generate manual thresholding standards for Figure 1, two independent reviewers established manual thresholds by demarcating (to the best of their ability) cell borders in each image. Results from the quantitative assessment of the different thresholding methods described were compared with each of the reviewers' images and the results were averaged. (TIFF) [file pone.0060988.s001.tiff]

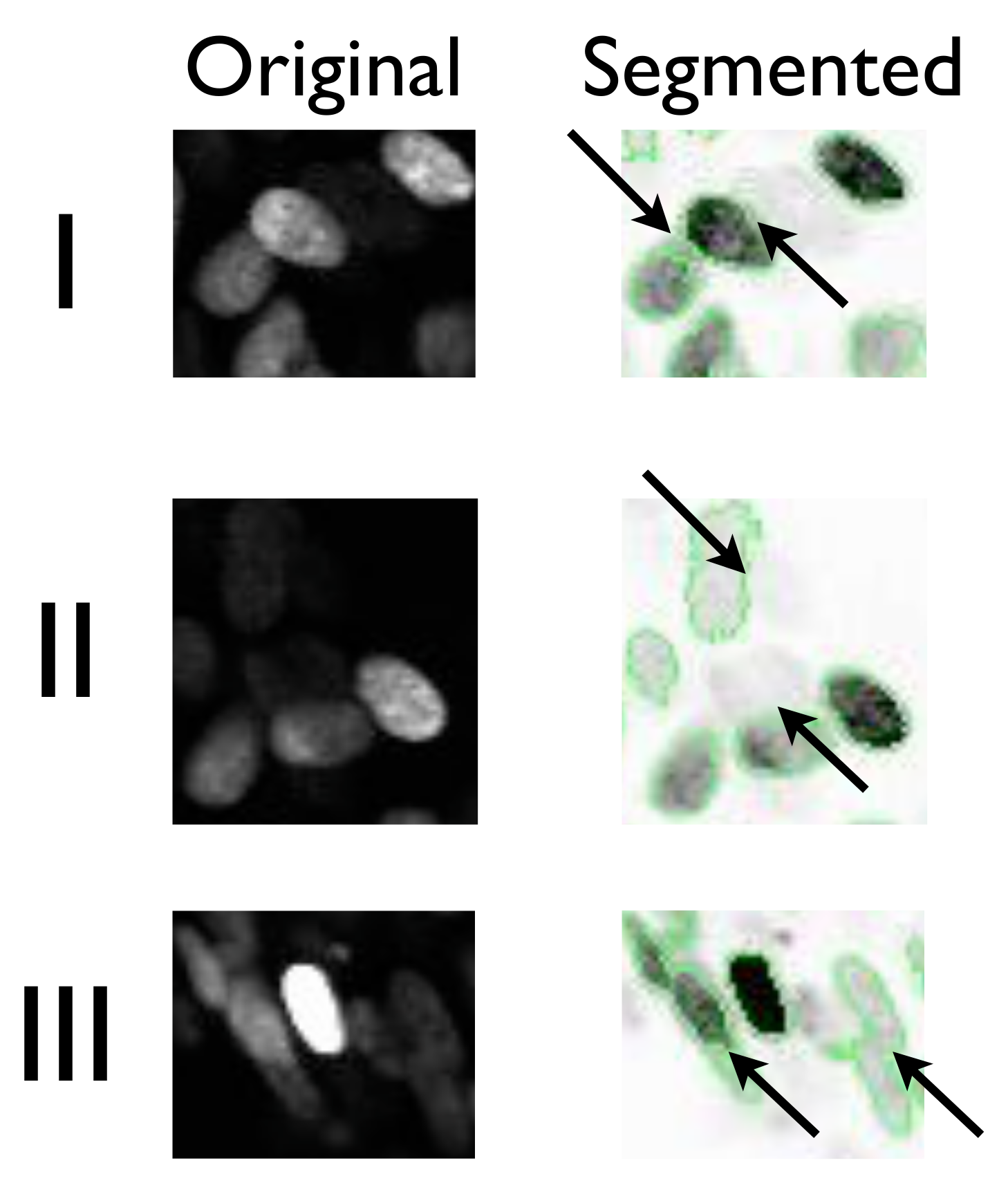

Supplement: Figure S2 — Additional typical intravital images used to perform ranking analysis in Figure 2D . Images were analyzed as described for those in Figure 1. (TIFF) [file pone.0060988.s002.tiff]

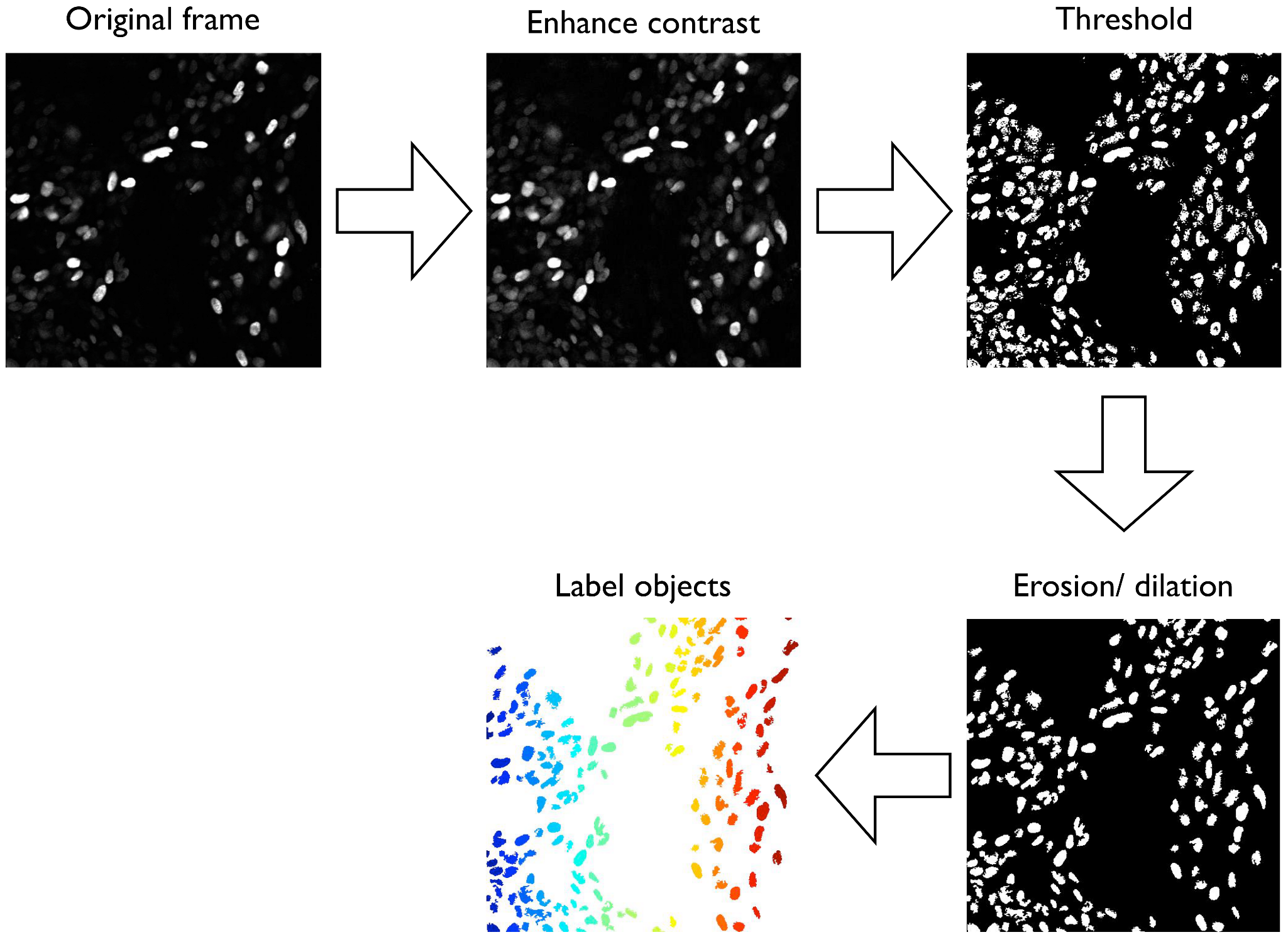

Supplement: Figure S3 — Detailed view of overall morphological operations and object labeling on an example image. This image was thresholded using Ray's method as described, followed by standard morphological operations to remove artifacts produced by the thresholding process. A rainbow color labeled image is presented to show distinct objects recognized by the analysis program. (TIFF) [file pone.0060988.s003.tiff]

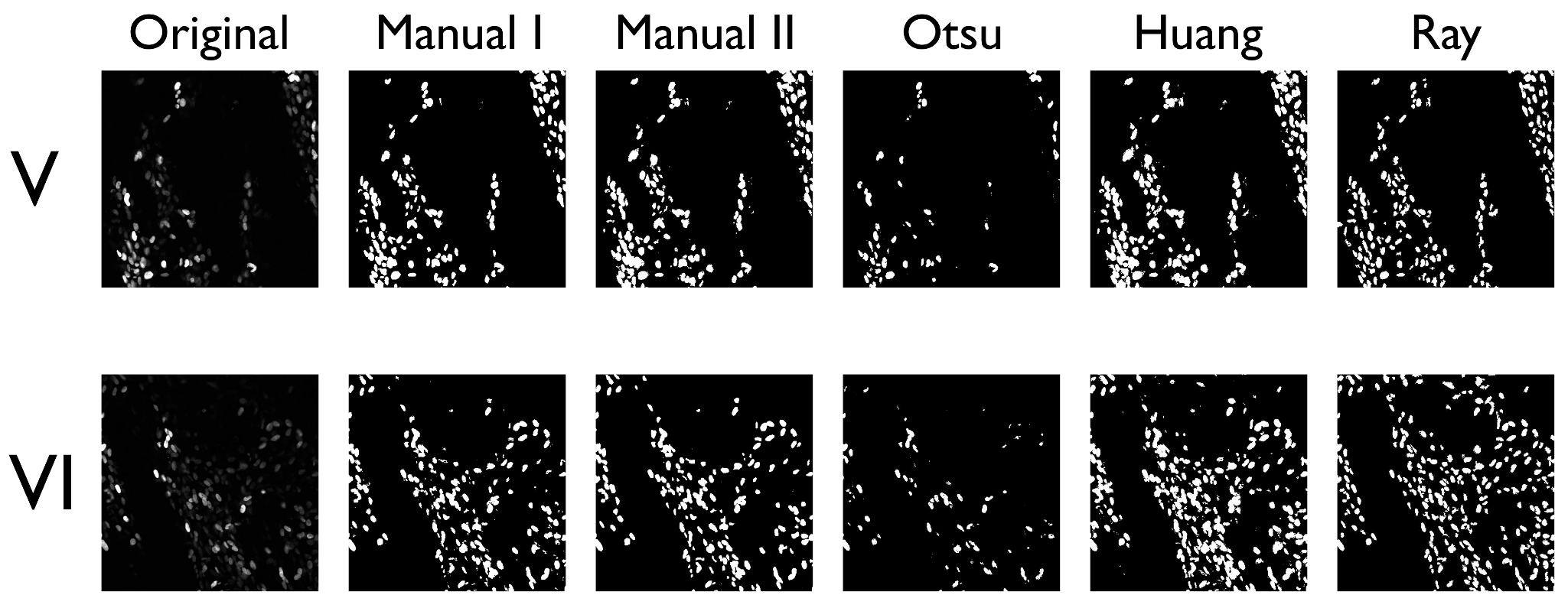

Supplement: Figure S4 — (I–III) Detailed views of cells with heterogeneous fluorescence (indicated by arrows) and segmentation of these areas via the reported algorithm. (TIFF) [file pone.0060988.s004.tiff]
